# Characterization and Validation of Flexible Dry Electrodes for Wearable Integration

**DOI:** 10.3390/s23031468

**Published:** 2023-01-28

**Authors:** Tiago Nunes, Hugo Plácido da Silva

**Affiliations:** 1 PLUX Wireless Biosignals, 1050-059 Lisbon, Portugal; 2 NOVA School of Science and Technology, NOVA University of Lisbon, 2829-516 Caparica, Portugal; 3 Instituto de Telecomunicações, 1049-001 Lisboa, Portugal

**Keywords:** carbon, dry electrodes, electrocardiogram, graphene, silver, wearables

## Abstract

When long-term biosignal monitoring is required via surface electrodes, the use of conventional silver/silver chloride (Ag/AgCl) gelled electrodes may not be the best solution, as the gel in the electrodes tends to dry out over time. In this work, the electrical behaviour and performance of dry electrodes for biopotential monitoring was assessed. Three materials were investigated and compared against the gold-standard Ag/AgCl gelled electrodes. To characterize their electrical behaviour, the impedance response over the frequency was evaluated, as well as its signal to noise ratio. The electrodes’ performance was evaluated by integrating them in a proven electrocardiogram (ECG) acquisition setup where an ECG signal was acquired simultaneously with a set of dry electrodes and a set of standard Ag/AgCl gelled electrodes as reference. The obtained results were morphologically compared using the Normalised Root Mean Squared Error (nRMSE) and the Cosine Similarity (CS). The findings of this work suggest that the use of dry electrodes for biopotential monitoring is a suitable replacement for the conventional Ag/AgCl gelled electrodes. The signal obtained with dry electrodes is comparable to the one obtained with the gold standard, with the advantage that these do not require the use of gel and can be easily integrated into fabric to facilitate their use in long-term monitoring scenarios.

## 1. Introduction

In recent years, there has been an increasing interest in the use of dry electrodes for biosignal acquisition systems. This is particularly noticeable in areas such as wearable technologies. Previous works have looked into dissimulating the device in everyday objects in an attempt to acquire biosignals in the least invasive way. These objects include computer keyboards, video game controllers, steering wheels in vehicles and even sanitary facilities [1,2,3], allowing for the off-the-person monitoring of electrocardiogram (ECG) signals. Regarding wearable applications, other works make use of body motion to control virtual or physical objects remotely. In [4], Tsuboi et al. developed a wearable controller in the shape of a glove that could be used to record electromiograms (EMG) using dry electrodes, aiming to simulate the movements of a hand in a virtual environment. Poon et al. [5] demonstrated how surgical robots’ interfaces can be improved by integrating EMG signals in the controls of the robot, leading to new human–computer interactions (HCI). A new HCI was also developed by Zhu et al. [6] where a motion-detection wearable interface could record the finger motions of the user with enough accuracy to control numerically operated machines or movable objects in virtual environments. This raised interest in the development of wearables, and the tendency to adopt dry electrodes in their manufacturing motivated us to conduct research on the validation of these devices against the established gold standard, using a device for ECG acquisition as our use-case. The development of ECG monitoring systems has been the major focus in many publication report works, using dry electrodes made from various materials [7,8,9,10]. This is no surprise, as cardiovascular diseases (CVD) are still the leading cause of death worldwide [11,12,13]. For more than 30 years, they have been the cause of more than 12 million deaths per year [14], with ischemic heart disease at the top of the table. Not only is ischemic heart diseases responsible for the most CVD-associated deaths, it also registered the highest increase in the last 20 years, with almost 9 million deaths in 2019 [15]. The diseases is more prone to affect the elderly [16], and in a world with an ever-aging population, it comes as no surprise that, without proper measures to prevent the disease effects, these numbers continue to increase [17,18]. Proper surveillance and early diagnosis can be a key factor in preventing severe cases of CVD and promoting a fast recovery for those affected by them. Made possible by advances in technology, remote monitoring and home-care solutions are increasingly being adopted by patients to continuously monitor their health condition [17], as well as by physicians in hospitals as a means of discharge in a post-intensive care recovery scenario [19]. For CVDs, the ability to record an electrocardiogram (ECG) with non-invasive methods and the possibility to detect heart abnormalities by analyzing its characteristics make the ECG a good candidate for evaluation of the heart condition [12,20,21,22,23].

Such a measurement can be taken at the skin surface by recording the biopotentials that originate from the heart and propagate to the skin surface by means of ionic currents [24]. These are measured using biopotential electrodes as a transducer; they convert an ionic current into an electron current [24,25,26]. The most commonly used type of electrode is silver/silver chloride (Ag/AgCl) gelled electrodes. Its simplicity of manufacturing, good signal quality and low cost make it the predilection for recording biopotentials at the skin surface. Nonetheless, these electrodes, also known as wet electrodes, present a few disadvantages that must be addressed. Whenever Ag/AgCl electrodes are used, skin preparation is necessary. In fact, the outer layer of the skin surface must be prepared by cleaning the dead cells, thus reducing its thickness and interface impedance [24]. Moreover, these electrodes are called ‘wet’ due to the necessity of applying an electrolyte-based gel with the purpose of further reducing skin–electrode impedance. The ions present in the electrolyte cause a voltage drop known as half-cell potential, which appears on the ECG signal as a DC offset. For continuous monitoring over large periods of time, the gel must be replaced as it dries, which affects the interface impedance to the detriment of the signal quality. Additionally, attention should be paid when using the electrolytic gel, as there are reports of adverse reactions to its use (dermatitis) [12,23,24,25,26,27,28]. Finally, this kind of electrode is kept in place on the body surface using adhesive tapes, which constrains the body’s movements and induces discomfort due to its bulky nature [23]. These drawbacks have increased interest in the use of dry electrodes. In fact, these do not require skin preparation, which is time-consuming, or the use of electrolytic gel, thus eliminating the problems associated with this [24,29]. Many different approaches have been used for dry electrode design. Some examples of these techniques are textile electrodes [30,31] silver ink [26,32,33] and carbon-based [34,35,36] techniques. In this paper, three new electrodes are compared with the most commonly used Ag/AgCl gelled electrodes. These are dry electrodes made of silver, graphene and carbon. They are printed on extremely thin substrates, which can be easily integrated into fabrics, facilitating their use in wearables or apparel for long-term monitoring. The remainder of this work starts with a characterization of the electrical properties of the electrodes, evaluating their impedance and Signal to Noise Ratio (SNR), followed by an evaluation of the acquired signal when incorporated in a biosignals’ acquisition device. Following this, the results of these measures are presented and discussed. In the case of the acquired ECG signals, some metrics are employed to evaluate and compare the performance of the electrodes being tested against the state of the art. The final conclusions of this work are presented in the final section.

## 2. Materials and Methods

**Figure 1:**
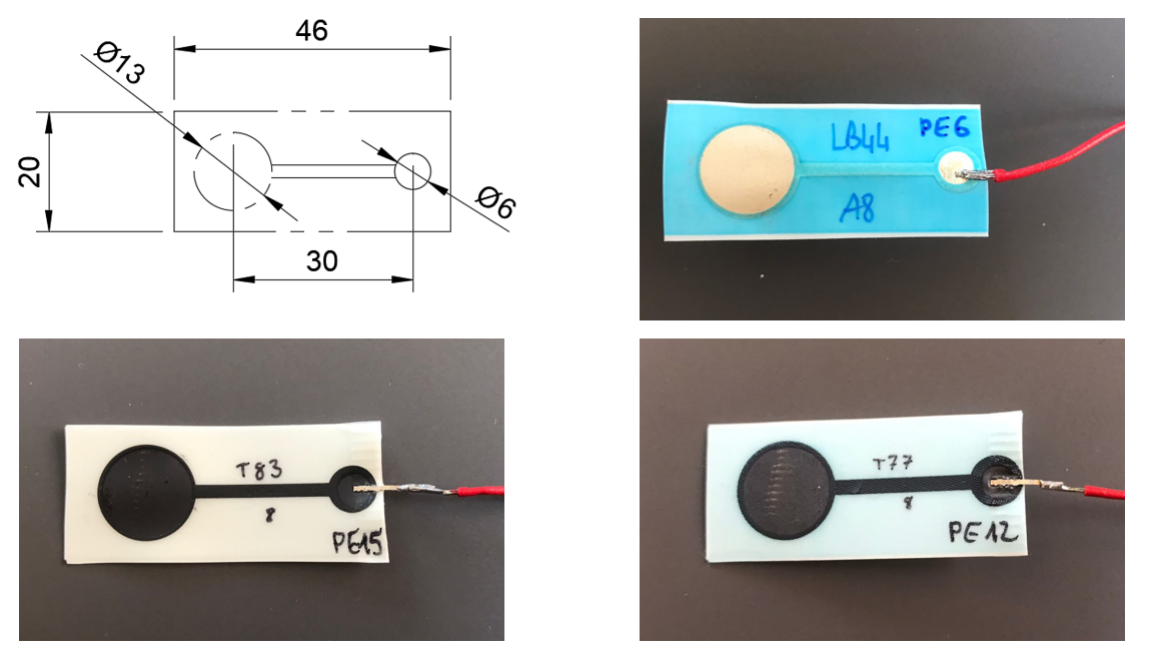
On the (**top**), from (**left**) to (**right**): electrodes’ dimensions in mm and silver electrode. On the (**bottom**), from (**left**) to (**right**): carbon and graphene.

As previously mentioned, the electrodes were manufactured on three different materials using the same printing and transferring technologies, developed by CEA LITEN—Innovation Laboratory for New Energy, Technologies, and Nanomaterials. All the sensors present the same form factor, as detailed in Figure 1 and are printed on an extremely thin and flexible film, allowing for the comfortable and versatile integration of these sensors in wearables. This can contribute to reducing the skin–electrode impedance and motion-related artifacts, as the electrodes better adapt to the body [37].

These electrodes will be evaluated in two main phases. First, the electrical behavior of each set of electrodes will be assessed. For this purpose, their impedance characteristics and signal to noise ratio will be evaluated. In the second phase, the electrodes will be integrated in an ECG acquisition setup and in vivo tests will be performed to compare their performance against the most commonly used Ag/AgCl wet electrodes.

### 2.1. Electrical Impedance

**Figure 2:**
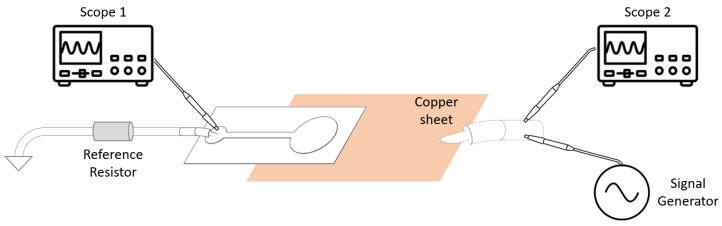
Impedance measurement circuit.

An impedance analyzer was used to evaluated the electrode impedance. The device used for this purpose was the Impedance Analyser tool of the Analog Discovery Digilent 2, which is capable of measuring the complex impedance of the device under test (DUT)-separating resistance from reactance and measuring the signal phase. Its main specifications are given in Table 1. The DUT was placed into a conditioning circuit according to the scheme in Figure 2.

The signal generator (Wavegen 1) applied the signal to the DUT through a copper sheet, whose resistance was negligible, and a probe measured this (Scope 2). A known reference resistor, of a similar order of magnitude as the DUT, was connected in series and the voltage across this was measured with a second probe (Scope 1). By measuring the voltage at both ends of the DUT, the impedance analyser was able to deduce the current flow, the voltage across, and the phase between the two. In these measurements, the signal generator was set to output a sine wave with 100 mV of amplitude (200 mV peak-to-peak), while the scope swept the frequencies from 1 Hz to 5 kHz, with 100 measurement points per decade, which were evenly distributed in a logarithmic scale.

### 2.2. Signal to Noise Ratio

Signal to Noise ratio is defined as the ratio between the signal amplitude and the noise amplitude, and is given in decibels. This can be expressed with Equation (1) [38,39,40].

(1) $$SNR_{dB}=10*log_{10}\frac{P_{signal}}{P_{noise}}=20*log_{10}\frac{V_{signal}}{V_{noise}}$$

To analyse the SNR of the electrode, an AC signal was applied across the sample using the signal generator embedded within the Analog Discovery 2 Digilent. The signal was then measured at the output of the signal generator and at the end of the electrode. By subtracting the first signal from the second, we removed the injected signal to obtain the noise induced by the electrode. This was achieved with the software associated with the scope, Waveforms, which performed the mathematical operation. The measuring apparatus is identical to the scheme in Figure 2.

### 2.3. In Vivo Acquisitions

**Figure 3:**
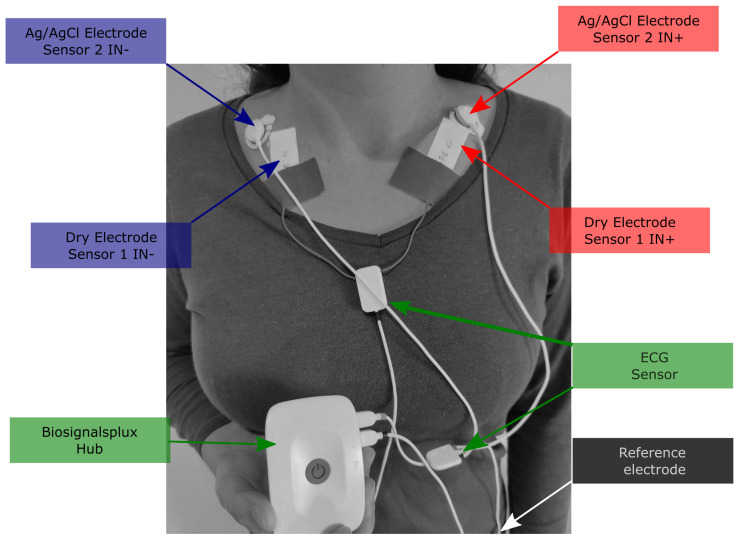
Electrodes’ placement.

For the in vivo acquisition, two sensors were used to acquire two ECG signals: one with dry electrodes and the other with gelled electrodes. Both were connected to the same acquisition device, a Biosignalsplux Hub (PLUX Biosignals, www.pluxbiosignals.com, accessed on 15 December 2022, Lisbon, Portugal). This is capable of acquiring physiological signals while streaming them to a nearby computer that can be used to visualize the acquisition in real-time and perform post-processing operations in the acquired signal. The ECG sensor has a bandwidth of 25–100 Hz [41]; therefore, a sampling rate of 400 Hz was used to sample the ECG signal. The first acquisition setup consisted of an ECG sensor using 3 Kendall H124SG Ag/AgCl electrodes, which was used as reference gold standard [42]. Figure 3 illustrates the positioning of the electrodes on the subjects. The positive and negative electrodes were placed on the left and right collar bones, respectively, while the reference electrode was placed on the iliac crest. The second acquisition setup used two dry carbon electrodes, placed on the collar bones next to the two gelled electrodes. These were positioned as close as possible, so that the two acquired signals could be compared with each other. A metallic contact forms the interface between the electrode and the wire that is connected to the sensor. This was insulated to avoid contact with the skin, which would compromise the acquired signal. Since both ECG sensors were connected to the same acquisition device, only one reference electrode was needed.

## 3. Results

The acquired signals were exported and further analyzed using MATLAB R2022a. To estimate the SNR of each type of electrode (silver, carbon and graphene), the signals obtained with the methods described in Section 2.2 were imported and the amplitude was calculated. Following Equation (1), the SNR was then deduced for each material, as summarized in Table 2.

**Figure 4:**
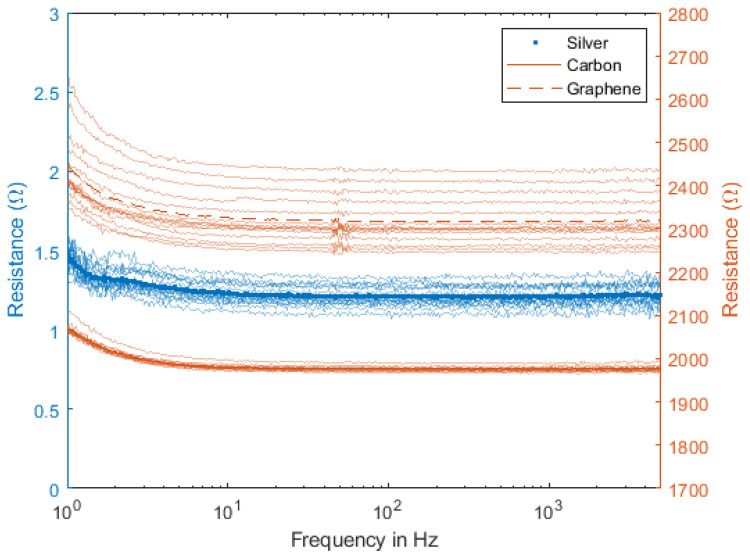
Impedance Response.

The impedance response of the materials was measured according to the methods described above, and the results are shown in Figure 4. If there is a clear difference between the mean nominal values of each material, with silver presenting values between 1 Ω and 2 Ω, carbon between 1800 Ω and 2000 Ω and graphene between 2200 Ω and 2500 Ω, the impedence value’s dependency on the frequency is clearly similar to every set of electrodes. This is especially noticeable in the range of frequencies below 100 Hz, where the impedance decreases with frequency. These results are in line with previous studies, which show that the electrical behavior of such electrodes is similar to a resistor in parallel with a capacitor: at lower frequencies, the overall impedance is set by the resistor alone, whereas, in the upper spectrum, the capacitor effect takes over, thus reducing the value of the global impedance [11,26,40,43,44,45]. This is particularly relevant for most biosignals, where the bandwidth of the signals under study is below 200 Hz [46,47].

To analyze the in vivo acquisitions made using the dry electrodes under test, two metrics were employed in the morphological comparison between the electrodes under test and the gold standard. For this purpose, two of the most common metrics were chosen: the Root Mean Square Error (RMSE) and the cosine similarity (CS) [48,49,50]. The RMSE is scale-dependent, as it directly compares the amplitude at each point in time got the two signals. For that reason, a normalization step is required, since the two signals were acquired with two different sensors. For this, a mean normalization was used, as defined by Equation (2) (where x is the signal and µ its mean value).

(2) $$x_{norm}\left[i\right]=\frac{x\left[i\right]-\mu_{x}}{\max(x)-\min(x)}$$

Once we normalize the values, we can calculate the RMSE. It is worth noting that, since we have normalized the values, this metric will give us the normalized RMSE. This is defined by Equation (3) (x and y are the two signals being compared with length n):

(3) $$\mathrm{nRMSE}\left(x,y\right)=\sqrt{\frac{\sum_{k=1}^{n}{\left(x_{norm}\left[k\right]-y_{norm}\left[k\right]\right)}^{2}}{n}}$$

The CS is defined between −1 and 1. As it approaches 1, it indicates high similarities between the two signals (with 1 being perfect similarity); when the value is close to 0, it indicates no similarity, while a value close to −1 indicates a highly negative similarity (or symmetry). Contrarily to the RMSE, the CS is not sensitive to the magnitude, but rather to the ’angle’ between the two signals: it compares the shape of the signals. Furthermore, it is sensitive to time shifts and can, therefore, be used to access how synchronized the signals are.

(4) $$CS=\frac{\sum_{i=1}^{n}x_{i}y_{i}}{\sqrt{\sum_{i=1}^{n}x_{i}^{2}}\sqrt{\sum_{i=1}^{n}y_{i}^{2}}}$$

**Figure 5:**
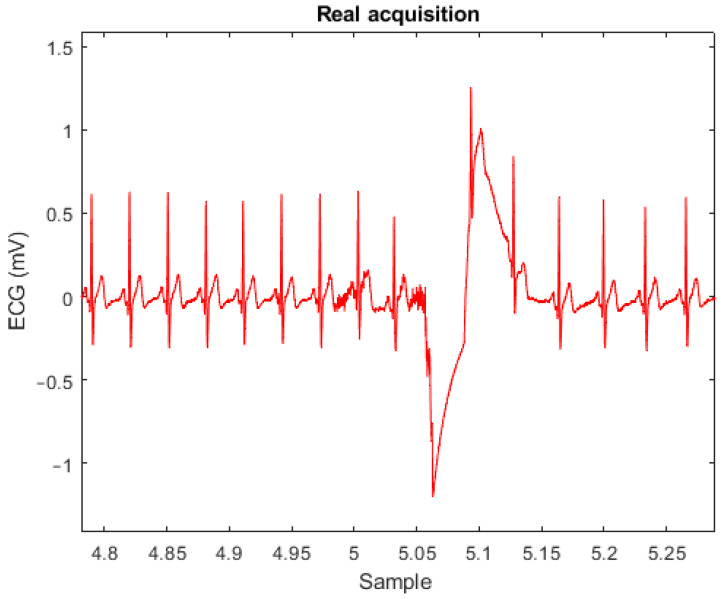
Artifacts present during a regular acquisition.

**Figure 6:**
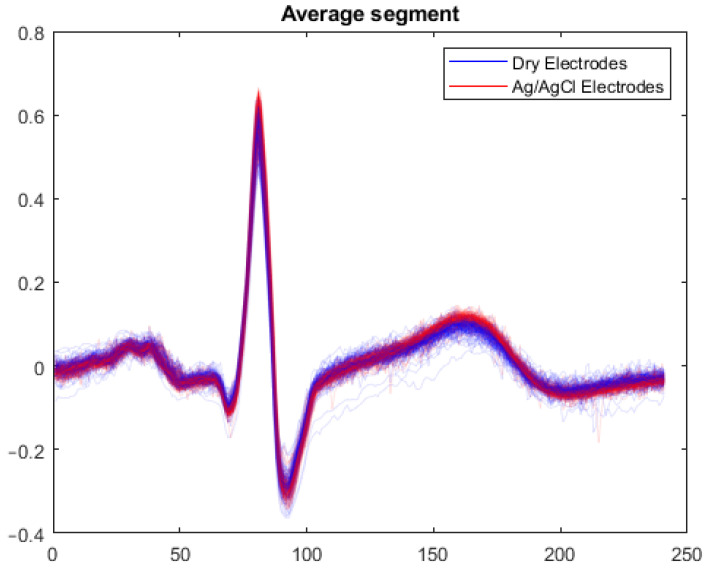
Beat-by-beat comparison.

Before applying these metrics to the signals acquired, they were segmented in portions to remove the largest artifacts present, due to factors such as the positioning of the electrodes, accidental touches, body movements and others. An example of the artifacts present during an acquisition is presented in Figure 5. Then, the segments were further sampled beat-by-beat. This was carried out by detecting the R-peaks of the QRS complex in each segment and then applying a window of 600 ms around the peak (200 ms before and 400 ms after). Extracting the QRS complex by windowing it is necessary to perform the above-mentioned morphological comparisons with all the important ECG features used for the diagnosis of different arrhythmia (R-R interval, S-T segment, T wave amplitude, …) [51,52]. In Figure 6, the superposed QRS complexes extracted according to this methodology show how the dry electrodes are able to give the same average signal as the gelled electrodes without compromising the small features. The RMSE and CS were then calculated for each beat, providing a beat-by-beat comparison of the obtained signals. The results obtained by these metrics are summarized in Table 3.

Presenting the lowest RMSE value and the highest CS value, the silver electrodes seem to have the best results; however, the other materials should not be disregarded, as their values are very close. Some structural modifications worth mentioning occurred on the graphene electrodes as they became brittle with time, and silver electrodes gained a thin layer of silver oxyde on their surface, changing and degrading its impedance.

## 4. Conclusions

This paper proposed evaluating and characterize three different dry electrode materials, made from silver, carbon and graphene, as an alternative to the commonly used gold-standard Ag/AgCl gelled electrodes. These are intended to respond to the increased need and demand for comfortable and wearable materials for the long-term monitoring of biosignals. Electrical impedance and signal to noise ratio were evaluated, and in vivo tests were performed. Results showed a relationship between impedance and frequency, especially at low frequencies. This calls for further research into the behavior of these electrodes in this range of values, as the biosignals acquired with skin electrodes are predominantly present at these frequencies (electroencephalogram, electrodermal activity, electrocardiogram, electromyogram). The in vivo tests showed a good overall performance, with an RMSE lower than 0.03 and a CS as high as 0.9933 ± 0.0068 in the case of silver electrodes. These values were obtained after excluding the motion artifacts, which were greater with the dry electrodes than with the gelled Ag/AgCl. This is due to the fact that, as the gold-standard was adhesive, it could maintain a better stability with regard to the contact point. Therefore, by guaranteeing a good stability for the dry electrode on the skin, it is safe to say that these electrodes are good candidates to replace the common Ag/AgCl wet electrodes on applications requiring long periods of constant monitoring.

## Figures and Tables

**Table 1:** Analog Discovery Digilent 2 Specifications.

Analog Discovery Digilent 2	
Bandwidth	30 MHz
Resolution	14-bits
Input Impedance	1 MΩ
Sampling rate	100 MS/s

**Table 2:** Signal to Noise Ratio.

Electrode Material	SNR(dB)
Silver	52.01 ± 4.86
Graphene	48.96 ± 2.52
Carbon	47.34 ± 2.84

**Table 3:** RMSE and CS for all beats compared.

Material	RMSE	CS	# Beats
Silver	0.0135±0.0048	0.9933±0.0068	2522
Graphene	0.0268±0.0101	0.9683±0.0316	2185
Carbon	0.0242±0.0081	0.9764±0.0196	1288

## Data Availability

Not applicable.
